# Determining Factors Affecting the Acceptance of Medical Education eLearning Platforms during the COVID-19 Pandemic in the Philippines: UTAUT2 Approach

**DOI:** 10.3390/healthcare9070780

**Published:** 2021-06-22

**Authors:** Yogi Tri Prasetyo, Ralph Andre C. Roque, Thanatorn Chuenyindee, Michael Nayat Young, John Francis T. Diaz, Satria Fadil Persada, Bobby Ardiansyah Miraja, Anak Agung Ngurah Perwira Redi

**Affiliations:** 1 School of Industrial Engineering and Engineering Management, Mapua University, 658 Muralla St., Intramuros, Manila 1002, Philippines; racroque@mymail.mapua.edu.ph (R.A.C.R.); thanatorn@webmail.npru.ac.th (T.C.); mnyoung@mapua.edu.ph (M.N.Y.); 2 School of Graduate Studies, Mapua University, 658 Muralla St., Intramuros, Manila 1002, Philippines; 3 Logistics and Supply Chain Management Program, Nakhon Pathom Rajabhat University, Nakhon Pathom 73000, Thailand; 4 Department of Finance and Accounting, Asian Institute of Management, 123 Paseo de Roxas, Legazpi Village, Makati 1229, Philippines; jdiaz@aim.edu; 5 Department of Business Management, Institut Teknologi Sepuluh November, Kampus ITS Sukolilo, Surabaya 60111, Indonesia; satriafp@gmail.com (S.F.P.); bobard.m@outlook.com (B.A.M.); 6 Industrial Engineering Department, BINUS Graduate Program—Master of Industrial Engineering, Bina Nusantara University, Jakarta 11480, Indonesia; wira.redi@binus.edu

**Keywords:** UTAUT2, structural equation modeling, medical education, eLearning platform

## Abstract

eLearning has been the medium of delivery of medical educational institutions to address the scarcity of medical professionals during the COVID-19 pandemic. In this study, the Unified Theory of Acceptance and Use of Technology (UTAUT2) was extended to determine the factors affecting the acceptance of eLearning platforms to medical education in the Philippines during the COVID-19 pandemic. A total of 360 medical students voluntary participated and answered an online questionnaire that consisted of 40 questions. Structural Equation Modeling (SEM) indicated that performance expectancy was found to have the highest effect on behavioral intention, which was followed by learning value and instructor characteristics. A high behavioral intention was found to affect the actual use of eLearning platforms. Interestingly, social influence and habit were found not to be significant to behavioral intentions. This study is the first study that has explored the acceptance of eLearning platforms among medical students in the Philippines during the COVID-19 pandemic. The findings can be a theoretical guideline of the Commission on Higher Education of the Philippines for eLearning platforms. Finally, the framework would be very valuable for enhancing the open innovation in eLearning platforms in medical fields worldwide.

## 1. Introduction

Distance learning has been implemented in the education sector due to the COVID-19 pandemic [1,2,3,4]. This type of learning has been widely utilized by almost 1.6 billion students or 94% of the world’s student population in more than 190 countries [4]. In a developing country like the Philippines, over 28 million students across all levels resumed classes [5], and it was delivered through a mix of modular learning, TV or radio broadcasts [6], and even through learning management systems. 

Learning management systems (LMS) consist of eLearning platforms [7]. It is a versatile and economic method of delivering education [7,8]. LMS enhances teaching and learning in higher education with the use of asynchronous and synchronous communication channels, provisioned online content, and interactive assessment tools [9]. The success of an LMS implementation is based on the understanding of the factors that can impact the intention of students towards LMS and its usage [9]. 

Previously, there were several studies related to LMS worldwide. In the University of Malaya—Malaysia, a Unified Theory of Acceptance and Use of Technology (UTAUT2) approach was utilized to evaluate the LMS [9]. The results showed that performance expectancy, social influence, and learning value had significant impacts on the behavioral intentions towards LMS [9]. In Hong Kong, UTAUT2 was also utilized to analyze consumer acceptance and the use of information technology [10]. Other studies utilized the Technology Acceptance Model (TAM) to analyze eLearning acceptance [11,12]. The findings indicated that computer self-efficacy, an individual’s perception on his or her ability to use computers given a specified task, had a significant impact on the ease of use of eLearning systems [11,12]. In addition, perceived ease of use also had a significant effect on the intention to use the e-learning platform [11].

According to the World Health Organization (WHO), eLearning is capable of supplying the estimated 4.3 million global shortage in health workers [13]. In the Philippines, a recent study of the University of the Philippines Population Institute (UPPI) and Demographic Research and Development Foundation, Inc. (DRDF) highlighted the shortage of healthcare workers, namely doctors, nurses, and midwives, even prior to the COVID-19 pandemic [14]. Their study aimed to provide a guideline in formulating a human resource policy for health, which is critical in containing the pandemic [14]. 

Despite having one of the strictest and longest lockdowns in the world [15], the Professional Regulation Commission of the Philippines (PRC) and the Professional Regulatory Board of Medicine recognize the urgent need for physicians for continuing medical education [16]. However, no study has been conducted so far particularly related to eLearning for medical students in the Philippines. Moreover, due to the sudden quarantine measures imposed by the government, medical schools only had a short time frame to restructure their curriculum, train faculty, and prepare students for eLearning [17]. However, PRC still proceeded with the continuation of the physician licensure examination (PLE) and qualifying assessment for foreign medical professionals (QAFMP) [16]. To suffice for the current gap in healthcare workers, eLearning platforms were put in place by Higher Educational Institutions (HEIs) to continue training and educating students to become professionals. 

This study aims to determine the factors affecting the acceptance of medical education eLearning platforms (e.g., Moodle, Docebo, and Blackboard) during the COVID-19 pandemic by utilizing the UTAUT2 approach. This study is the first study that has explored the acceptance of eLearning platforms among medical students in the Philippines during the COVID-19 pandemic. The findings can be the theoretical guidelines of the Commission on Higher Education of the Philippines for enhancing eLearning platforms. Finally, the framework would be very valuable for enhancing open innovation in the eLearning platforms in medical fields worldwide.

## 2. Conceptual Framework

**Figure 1:**
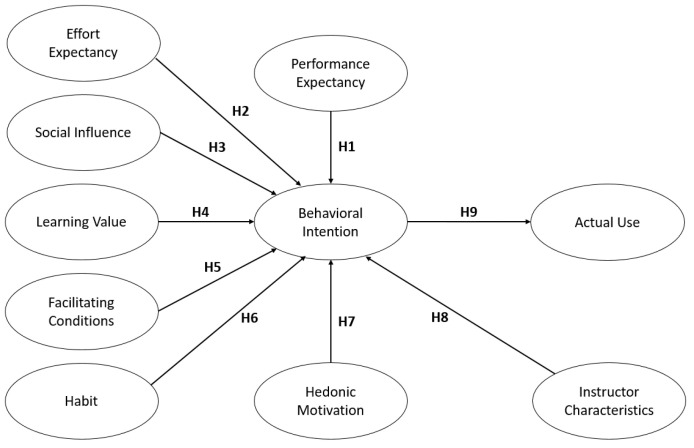
The proposed conceptual framework.

Figure 1 represents the proposed conceptual framework of the study. Supported by some studies [9,10,11], the proposed framework was based on the UTAUT2, with two additional variables: Learning Value and Instructor Characteristics. UTAUT2 was utilized, since this theory is one of the most widely utilized theories in the context of technology acceptance. 

Performance Expectancy (PE) is the extent or level of belief of an individual that utilizing the eLearning platform provides benefits in performing different activities [9,18,19,20]. It pertains to the perceived performance improvement of the individual by using the technology [21]. It is considered to be similar to perceived usefulness in the Technology Acceptance Model or (TAM) [20]. PE is proved to have a strong influence towards intention to use [20]. As such, we hypothesized that:

**Hypothesis** **1** **(H1).** *Performance expectancy has a positive influence on a student’s behavioral intentions in using the eLearning platform*.

Effort Expectancy (EE) is the extent or level of ease associated with the use of the system [19]. EE has been proven to have considerable influence on the intention to use information and communication technologies (ICT) or, in this case, eLearning platforms. [10,20]. We hypothesized that:

**Hypothesis** **2** **(H2).** *Effort expectancy has a positive influence on a student’s behavioral intentions in using the eLearning platform*.

Social Influence (SI) is defined as the degree to which an individual perceives that others, especially other students, faculty members, and family, should use the eLearning platform [20]. From this, we formulated the hypothesis:

**Hypothesis** **3** **(H3).** *Social influence has a positive influence on the behavioral intentions to use the eLearning platform*.

Learning Value (LV). Venkatesh et al. [19] used price value [10] in their study. However, from a student’s perspective, the value is associated with the learning gained or achieved benefit from the eLearning platform. The students do not directly pay any cost-to-gain benefits from the use of LMS technology or eLearning platforms [9], because it is the institution that contracts eLearning companies for this. In some institutions, they have created their own platforms for students. Thus, learning value is defined as the student’s perception that the time and effort put in for learning represents a good value [9]. From this, we hypothesized:

**Hypothesis** **4** **(H4).** *Learning value has a positive influence on the behavioral intentions to use the eLearning platform*.

Facilitating Conditions (FC) is the accessibility of enough resources and support for an individual’s utilization of the technology [9]. Resources are the technical infrastructures that are in place to help students use the system such as internet connection and laptops/desktop [20]. According to Venkatesh et al. [19], facilitating conditions (FC) does not influence behavioral intentions (BI) because of the presence of effort expectancy (EE) in the model [9,19]. However, in reference to the model structured by Ain et al. [9], this research hypothesized:

**Hypothesis** **5** **(H5).** *Facilitating conditions has a positive influence on the behavioral intentions to use the eLearning platform*.

Habit (HB) is the habitual or automatic behavior towards using eLearning platform technology [9,18]. It presents the results of previous experiences [19,20]. Once a behavior becomes a habit, it is automatic and is performed without conscious decision [20,21]. For this, we hypothesized that:

**Hypothesis** **6** **(H6).** *Habit has a positive influence on a student’s behavioral intentions towards the eLearning platform.*

Hedonic Motivation (HM) is the fun or pleasure derived from using the eLearning platform [10]. It also pertains to perceived enjoyment in information systems (IS) and has been discovered to have a direct influence on the use of technology [9,10]. As such, we hypothesized that:

**Hypothesis** **7** **(H7).** *Hedonic motivation has a positive influence on the behavioral intentions to use the eLearning platform*.

Instructor Characteristics (IC**)** is the degree to which the teacher will have a concern, provide guidance, and accommodate their student’s needs [11]. According to Selim, H. M. [22], one of the significant indicators was the instructor’s attitude towards e-learning technology. Thus, we formulated the following hypothesis:

**Hypothesis** **8** **(H8).** *Instructor Characteristics has a positive influence on the behavioral intentions towards the eLearning platform*.

Behavioral Intention (BI) is the degree of the student’s intentions to use the eLearning platform in the future [18]. According to Ain et al. [9], numerous studies have reported that behavioral intentions significantly impact actual system use.

**Hypothesis** **9** **(H9).** *Behavioral intention has a positive influence on the actual use of the eLearning platform (US)*.

## 3. Methodology 

### 3.1. Participants

A total of 360 medical students participated in this study. They were taking the Doctor of Medicine program at accredited medical educational institutions in the Philippines that utilize eLearning platforms. Prior to the data collection, respondents were notified of confidentiality in each questionnaire. The institutional review board was waived, since this study mainly focused on the acceptance of medical education eLearning rather than human performance. Due to the COVID-19 pandemic, the data was gathered through a survey questionnaire created using Google forms.

Table 1 represents the descriptive statistics of the respondents. The majority of the respondent population was from the 18 to 34-year-old age group. Table 1 also indicates that a large portion of the respondents were daily users of their institution’s respective eLearning platform. The highest percentage of users showed that they spent more than 4 h using the platform.

### 3.2. Quistionnaire

The questionnaire consists of two parts. The first part contains the demographic profile questions, namely gender, age, year level, usage frequency, and the time spent using an eLearning platform. The second part has 40 items or indicators for the 10 latent variables, which were measured on a 7-point Likert Scale. The Unified Theory of Acceptance and Use of Technology 2 (UTAUT2) was the theoretical framework used for the questionnaire. The indicators (questions) were developed based on previous studies (See Appendix A) [9,11,12,20,23,24]. The 7-point Likert Scale was structured as follows: 1—Strongly Disagree, 2—Disagree, 3—Somewhat Disagree, 4—Neither Agree nor Disagree, 5—Somewhat Agree, 6—Agree, and 7—Strongly Agree UTAUT2.

### 3.3. Structural Equation Modeling

Structural Equation Modeling (SEM) is one of the multivariate analysis methods that is widely utilized to link several factor constructs simultaneously [20,21]. In the context of UTAUT2, this statistical approach was widely utilized instead of hierarchical regression. AMOS version 21 with the Maximum Likelihood Estimation (MLE) approach was utilized to run the SEM. 

Several indicators to justify the model fit were utilized: Goodness of Fit Index (GFI), Incremental Fit Index (IFI), Tucker Lewis Index (TLI), Comparative Fit Index (CFI), and Root Mean Square Error of Approximation (RMSEA). For GFI, IFI, TLI, and CFI, a value of higher than 0.8 was considered good, while, for RMSEA, a value of lower than 0.7 was considered good [25,26,27]. 

## 4. Results 

**Figure 2:**
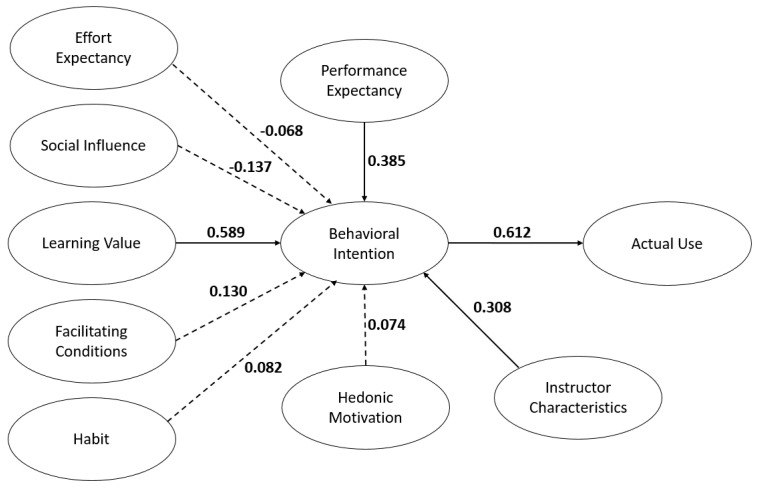
The initial model.

**Figure 3:**
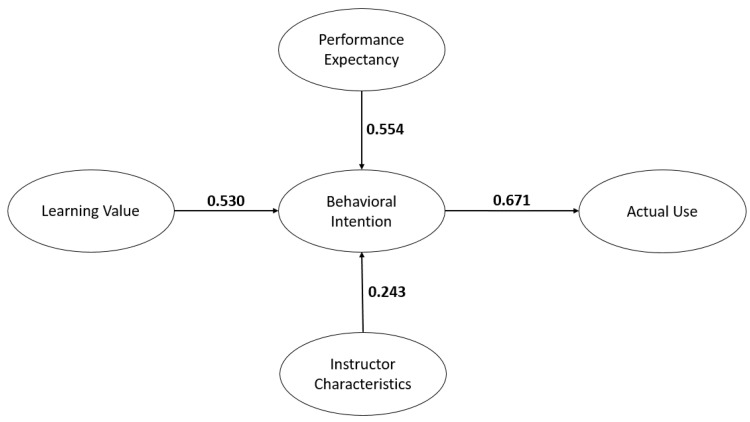
The final model.

Figure 2 demonstrates the initial model of the study. Unfortunately, several paths were found not to be significant: EE→BI, SI→BI, FC→BI, HB→BI, and HM→BI (Table 2). In SEM, statistical tests define the adequacy of model fit. We assessed how well the UTAUT2 theory fit the data gathered [26,28]. With prominent consideration of a theory, the most common change would be the deletion of a latent that does not meet the model fit or construct validity [26]. Thus, a revised final model was derived by eliminating these paths [29]. Figure 3 represents the final model of the study. In addition, Table 3 also represents the model fit of the final model. 

Table 4 shows the construct validity and reliability of the final model. According to Hair et al. [26], the factor loading exceeding 0.7 is an indication of a well-defined model structure; however, a value higher than 0.5 is still considered significant. Our results showed that all the factor loadings were higher than 0.5, implying that the indicators construct was a good representation of the selected latent variables. 

Subsequently, Table 4 also shows that the values of the average variance extracted (AVE) of the latent variables were higher than 0.5, except for facilitating conditions (FC). An AVE greater than 0.5 suggests adequate convergence, which means the indicators were closely related to the latent variable [26]. Finally, the construct reliability (CR) values of the latent variables were higher than the benchmark value of 0.7 [9]. According to Hair J. et al. [26], a high construct reliability demonstrates that the indicators represent the latent construct.

The relationships between the constructs of the final structural model were evaluated based on their level of statistical significance (*p*-value < 0.05) and their standardized loadings. Table 5 exhibits the direct, indirect, and total effect relationships of the latent variables. For the direct effects, Performance Expectancy was found to have the highest positive effect on Behavioral Intention (β = 0.554, *p* ≤ 0.001), followed by Learning Value (β = 0.530, *p* ≤ 0.001) and Instructor Characteristics (β = 0.243, *p* ≤ 0.001). The direct path of Behavioral Intention to Usage had a β-value of 0.671 with a statistical significance of less than 0.001. For the indirect effect towards the usage or use of the eLearning platform, Performance Expectancy had the highest effect on Usage (β = 0.372, *p* = 0.001), followed by Learning Value (β = 0.355, *p* = 0.001) and Instructor Characteristics (β = 0.142, *p* = 0.009). 

## 5. Discussion

eLearning has been the medium of delivery of medical educational institutions to address the scarcity of medical professionals [30,31,32]. In this study, the Unified Theory of Acceptance and Use of Technology (UTAUT2) was extended to determine the factors affecting the acceptance of eLearning platforms for medical education in the Philippines during the COVID-19 pandemic. 

Performance Expectancy was found to have a significant and positive effect on the intention to use the eLearning platform. The usefulness, the perceived accomplishment, the perceived productivity, and the perceived achievements were significant indicators that led to the behavioral intentions. Interestingly, PE was also found as the strongest predictor of BI. This finding was supported by Venkatesh [19], who stated that performance expectancy has consistently been a strong predictor of behavioral intention. Similarly, Dečman [32] also mentioned that it is the most significant construct in the acceptance of eLearning and institutions should examine this closely. Thus, medical institutions need to enhance the performance of the eLearning platform to enhance its utilization among medical students. 

Learning Value was found to have a positive effect on Behavioral Intention. The values such as worthiness, flexibility, usefulness, and well content of the eLearning platform were the key indicators that led to the behavioral intentions. These findings were consistent with Ain et al. [9], who also mentioned that the perceived value was the key for utilizing the eLearning platform. 

Instructor Characteristics was found to have a positive effect on Behavioral Intention. Instructor attitudes, such as being able to fully utilize the eLearning platform, keep answering questions, keep encouraging and motivating, were some key indicators that led to the utilization of the eLearning platform among medical students. These findings were also supported by Azizi [28], who also found that IC is a significant predictor of the intention to use the eLearning platform. Similarly, Roman and Plopeanu [33] also stressed the importance of the role of professors in the success of the learning process. Pedagogical and psychological skills of instructors and their respective personalities affect student interactions. The role of educators is to understand the needs and motivations to maintain learner engagement [17]. 

In terms of Social Influence, it is interesting to mention that this latent variable was found to be not a significant predictor for Behavioral Intention. This is contradictory, given the promotion of the government to distance learning [34] and the high global ranking of Filipinos in terms of internet and social media usage [35]. If proven significant, this could have supported the statement of Venkatesh et al. [10] regarding persons belonging to a unique group or higher status in the community as significant drivers of the use of technology. This is also contrary to a previous study [9] wherein peers’ and instructors’ views on the eLearning platform are influential toward the intention to use this technology. However, in the study of Chipeva [20], social influence was found as a weak indicator in a certain country due to performance expectancy being the major indicator. 

Habit was not a strong predictor of the Behavioral Intention to use the eLearning platform. Ain et al. [9] also found insignificance in the habit–behavioral intention relationship. In addition, Goncalves [36] also stated that, once the use of technology is established as a routine, individuals are more inclined towards using it. The findings of Baticulon, R. et al. [17] asserted that the pandemic has caused psychological stress on students and resulted to difficulty in focusing on their studies. Mental stress may have had an impact on the habit formation of medical students during this pandemic.

Facilitating conditions was not found to have significant effects on behavioral intention. A valid reason may be that effort expectancy has the effect of capturing FC [19]. Tarhini [37] also encountered the same results contradictory to their expectations. However, the findings of Azizi et al. [28] and Ain et al. [9] proved that the availability of technological infrastructures plays a vital role in the intention to use eLearning. 

For Hedonic Motivation, it was found to have an insignificant effect on the intention to use eLearning. This is parallel with the findings of Ain et al. [9]. Students do not see the use of the eLearning platform to be fun and enjoyable. This may be explained by the role of the eLearning platform, which is focused on providing quizzes, assignments, and other course-related activities. 

The results for Effort Expectancy showed that it was not a strong predictor for the intention to use eLearning. This result was similar to the findings of Chipeva et al. [20]. Students are more inclined toward the usefulness of the platform and consider it as a noncomplex system [9]. However, Venkatesh [10,19] emphasized that EE is a key driver in using information and communication technologies, because it surpasses the complexity of the system.

Finally, Behavioral Intention was shown to be of high significance in determining the actual use of the eLearning platform. This is consistent with previous studies using the same theoretical framework [9,20,28]. BI is highly influential on the actual use, as conceptualized in the UTAUT2 theory of Venkatesh [10]. 

### 5.1. Practical Implications

There should exist a system to evaluate the quality of eLearning material through some form of feedback from participants [38]. According to Joaquin et al. [15], there is still inadequacy in the response of Higher Educational Institutions (HEIs) in the Philippines in terms of the technologies for delivering education. The Commission on Higher Education (CHED) guidelines on the Doctor of Medicine program have not yet set standards for online learning [17]. Thus, the government, administrators, and faculty members need to collaborate to enhance the performance of the eLearning platform to enhance its utilization among medical students. Given the situation, the chairman of CHED calls for unity among HEIs to overcome the challenges brought about by the COVID-19 pandemic as we transition to online learning [34]. This study provides a significant and timely contribution in terms of the technological reputation of HEIs [9], the perspective of the students, and the effectiveness of the eLearning platform.

### 5.2. Limitations and Future Research

Regardless of the substantial contributions of the study, there were several limitations of the current study. First, our study only utilized the UTAUT2 approach. In fact, there are many other methods or more advanced models, such as combining UTAUT2 with several theories in psychology [29]. Second, our sample size was mainly medical students. Future research to compare premedical, medical, and resident students would be a very promising topic. Last, we did not incorporate the effect of individual characteristics and culture. Future research to incorporate these two latent variables would be another promising topic. 

## 6. Conclusions

During the COVID-19 pandemic, eLearning has been widely utilized as the medium of instruction, including among medical students [30,31]. In this study, the UTAUT2 was extended to determine the factors affecting the acceptance of eLearning platforms (e.g., Moodle, Docebo, and Blackboard [39]) in medical education in the Philippines during the COVID-19 pandemic [40]. A total of 360 medical students voluntary participated and answered an online questionnaire, which consisted of 40 questions. 

Structural Equation Modeling (SEM) [41] highlighted that performance expectancy was found to have the highest effect on behavioral intention, which was followed by learning value and instructor characteristics. With these highlights, medical institutions in the Philippines need to enhance the performance of the eLearning, particularly when it comes to usefulness, perceived accomplishments, perceived productivity, and perceived achievements while using the eLearning platforms. In addition, the values such as the worthiness, flexibility, usefulness, and well content of the eLearning platforms were also highlighted as the key indicators that led to the behavioral intentions.

This study is the first study that explored the acceptance of eLearning platforms among medical students in the Philippines during the COVID-19 pandemic. These findings can be the theoretical guidelines of the Commission on Higher Education of the Philippines for enhancing eLearning platforms. Although this study only utilized the UTAUT2 approach with two additional variables: Learning Value and Instructor Characteristics, the framework would be very valuable for enhancing the open innovation in eLearning platforms in medical fields worldwide [42,43,44,45,46].

## Figures and Tables

**Table 1:** Descriptive statistics of the respondents (*N* = 360).

Measure	Value	*N*	%
Gender	Male	96	26.67%
	Female	264	73.33%
Age	18–24 years old	204	56.67%
	25–34 years old	150	41.67%
	35–44 years old	3	0.83%
	Above 54	3	0.83%
Year Level	1st Year	33	9.17%
	2nd Year	36	10.00%
	3rd Year	90	25.00%
	4th Year (Junior Internship)	186	51.67%
	5th Year (Senior Internship)	15	4.17%
Usage Frequency	1 to 2 times a month	6	1.67%
	3–6 times a month	18	5.00%
	7–12 times a month	24	6.67%
	More than 12 times.	57	15.83%
	Daily	255	70.83%
How much time do I usually spend using an eLearning platform?	Less than 1 h	78	21.67%
	1 to 2 h	105	29.17%
	3 to 4 h	51	14.17%
	More than 4 h	126	35.00%

**Table 2:** Model modification.

Hypothesis		Preliminary Model		Final Model	
		β	*p*-Value	β	*p*-Value
1	PE→BI	0.385	***	0.554	*
2	EE→BI	−0.068	0.321	-	-
3	SI→BI	−0.137	0.519	-	-
4	FC→BI	0.130	0.072	-	-
5	LV→BI	0.589	***	0.530	***
6	HM→BI	0.074	0.253	-	-
7	HB→BI	0.082	0.718	-	-
8	IC→BI	0.308	***	0.243	*
9	BI→US	0.612	***	0.671	***

Note: *** *p* < 0.001 and * *p* < 0.05.

**Table 3:** Final model fit.

Goodness of Fit Measures of the SEM	Parameter Estimates	Minimum Cut-Off	Recommended by
Goodness of Fit Index (GFI)	0.814	>0.80	[26,27]
Root Mean Square Error of Approximation (RMSEA)	0.062	<0.07	[26]
Incremental Fit Index (IFI)	0.897	>0.80	[26]
Tucker Lewis Index (TLI)	0.834	>0.80	[26]
Comparative Fit Index (CFI)	0.865	>0.80	[26]

**Table 4:** Construct validity and reliability.

Latent Variables	Items	Cronbach’s α	Factor Loadings	Average Variance Extracted (AVE)	Composite Reliability (CR)
PE	PE1	0.913	0.765	0.715	0.909
	PE2		0.803		
	PE3		0.903		
	PE4		0.902		
LV	LV1	0.879	0.561	0.565	0.835
	LV2		0.690		
	LV3		0.880		
	LV4		0.833		
IC	IC1	0.732	0.604	0.500	0.798
	IC2		0.649		
	IC3		0.728		
	IC4		0.827		
BI	BI1	0.958	0.875	0.770	0.930
	BI2		0.917		
	BI3		0.813		
	BI4		0.902		
US	US1	0.849	0.701	0.566	0.839
	US2		0.771		
	US3		0.761		
	US4		0.775		

**Table 5:** Path analysis.

Variables	Direct	*p*-Value	Indirect	*p*-Value	Total	*p*-Value	Results
PE→BI	0.412	0.001	No path	-	0.554	0.002	Supported
IC→BI	0.290	0.001	No path	-	0.243	0.010	Supported
LV→BI	0.619	0.001	No path	-	0.530	0.000	Supported
BI→US	0.626	0.001	No path	-	0.671	0.001	Supported
IC→US	No path	-	0.163	0.009	0.181	0.016	Supported
LV→US	No path	-	0.355	0.001	0.388	0.000	Supported
PE→US	No path	-	0.372	0.001	0.258	0.035	Supported

## Data Availability

The data presented in this study are available on request from the corresponding author.

## Appendix A. Instruments

**Construct**	**Code**	**Indicator**	**Source**
Performance Expectancy (PE)	PE1	1. I find eLearning useful for my studies.	[9]
	PE2	2. eLearning allows me to accomplish class activities more quickly.	
	PE3	3. eLearning increases my learning productivity.	
	PE4	4. Using eLearning increases my chances of achieving things that are important to me.	[20]
Effort Expectancy (EE)	EE1	5. eLearning is easy to use.	[9]
	EE2	6. Learning how to use the eLearning platform is easy for me.	
	EE3	7. My interaction with the platform is clear and understandable.	
	EE4	8. It is easy for me to become skillful at using eLearning.	[20]
Social Influence (SI)	SI1	9. My peers who influence my behavior think that I should use eLearning.	[9]
	SI2	10. My friends who are important to me think that I should use eLearning.	
	SI3	11. My instructors, whose opinions that I value, prefer that I use eLearning.	
	SI4	12. eLearning is a status symbol in my environment.	[20]
Facilitating Conditions (FC)	FC1	13. I have the resources to use eLearning.	[9]
	FC2	14. I have the knowledge to use eLearning.	
	FC3	15. A specific person (or group) is available to assist when difficulties arise with using the eLearning platform.	
	FC4	16. There is compatibility between the platform that I use.	[20]
Learning Value (LV)	LV1	17. Learning through eLearning is worth more than the time and effort given to it.	[9]
	LV2	18. eLearning gives me the opportunity to decide about the pace of my own learning.	
	LV3	19. eLearning gives me the opportunity to increase my knowledge and to control my success (e.g., via quizzes and assignments/assessments, etc.).	
	LV4	20. Learning content that I require can be provided by the eLearning platform.	[12]
Hedonic Motivation (HM)	HM1	21. Using eLearning is fun.	[9]
	HM2	22. I enjoy using eLearning.	
	HM3	23. Using the eLearning platform is very entertaining.	
	HM4	24. The use of the eLearning platform amuses me.	
Habit (HB)	HB1	25. The use of eLearning has become a habit for me.	[9]
	HB2	26. I am addicted to using eLearning to accomplish my study tasks.	
	HB3	27. I must use eLearning for my studies.	
	HB4	28. Using eLearning has become natural for me.	
Instructor Characteristics (IC)	IC1	29. I feel the instructor is keen that we use e-learning-based units.	[11]
	IC2	30. We are invited to ask questions/receive answers.	
	IC3	31. The instructor encourages and motivates me to use the eLearning platform.	
	IC4	32. The instructor is active in teaching me the course subjects via the eLearning platform.	
Behavioral Intention (BI)	BI1	33. I intend to continue using eLearning.	[9]
	BI2	34. For my studies, I would use the eLearning platform.	
	BI3	35. I will continue to use eLearning on a regular basis.	
	BI4	36. I will recommend other students to use the eLearning platform.	[24]
Usage (US)	US1	37. I use the eLearning platform frequently during my academic period.	[9]
	US2	38. I use many functions of eLearning (e.g., discussion forums, chat sessions, messaging, downloading course contents, uploading assignments, etc.).	
	US3	39. I depend on the eLearning platform.	
	US4	40. I use the eLearning platform as a reference tool for my studies.	
